# Pharmacogenetic intervention in the Child and Adolescent Autism Day Therapeutic Unit

**DOI:** 10.1192/j.eurpsy.2024.623

**Published:** 2024-08-27

**Authors:** A. Alvarez, N. Santamaria, V. Bote, R. Medina, B. Sanchez, I. Mendez, J. A. Monreal, M. J. Arranz, A. Hervas

**Affiliations:** ^1^University Hospital Mutua Terrassa, Terrassa, Spain; ^2^Mental Health, University Hospital Mutua Terrassa; ^3^Mutua Terrassa Foundation, Terrassa, Spain

## Abstract

**Introduction:**

The ASD Therapeutic Day Unit is a tertiary care unit that consists of 20 beds, designed to facilitate the evaluation and treatment of children and adolescents with ASD who present high psychiatric comorbidity with behavioral problems, communication/language problems, sensory, and/or in the management of their repetitive and restricted interests. In addition to diagnosis and genetic counseling and clinical care, we offer the possibility of performing an individualized pharmacogenetic study in order to offer appropriate pharmacological treatment to patients with ASD and comorbidities.

**Objectives:**

The objective is to promote pharmacological tolerability, avoid unwanted side effects, as well as avoid the use of polypharmacy, in children with a tendency to poor drug metabolism.

**Methods:**

A review of the medical history of the patients included in the Blood Extraction Program of the ASD Day Therapeutic Unit is carried out during the year 2022. The existing medications at admission, the results of the pharmacogenetic analyzes carried out, and the pertinent changes in the pharmacological treatment of these children.

**Results:**

37 children were included in the program during 2022. The genes CYP1A2, CYP2C19, CYP2D6, CYP3A4 and 5-HTT were analyzed. The variant studied is described, as well as the observed genotype and the expected phenotype.

Of the 37 patients, 11 maintained the same pharmacological treatment as at the beginning of admission, 5 were not taking pharmacological treatment and 25 underwent a treatment modification.

The most frequently modified treatment was risperidone with aripiprazole (n=10), secondly risperidone with guanfacine (n=5), and thirdly fluoxetine with aripiprazole (n=2).

Furthermore, the degree of pharmacological polytreatment was reduced. 18 patients switched to a single drug, instead of 14. 11 patients 2 drugs (instead of 14), 3 patients 3 drugs instead of 4 and 5 patients remained without drug treatment.

**Conclusions:**

Patients with ASD have worse tolerability to pharmacological treatments than other patients with severe mental disorders.

The use of pharmacogenetics allows improving the cost/effectiveness of medical prescription, avoiding undesirable side effects or lack of effectiveness in the treatment of patients with ASD.

Promoting the implementation of pharmacogenetics in patients with ASD (among others) would improve the clinical situation of these patients more effectively and would improve the economic expenditure derived from erroneous prescription and/or excessive polypharmacy.

**Disclosure of Interest:**

None Declared

